# A Systematic Critical Appraisal of Clinical Practice Guidelines for Non‐Pharmacological ‎Conservative Management of Complex Regional Pain Syndrome (CRPS) Using the ‎AGREE‐II Instrument

**DOI:** 10.1111/jep.70417

**Published:** 2026-05-04

**Authors:** Erfan Shafiee, Joy MacDermid, Tara Packham, Ruby Grewal, David Walton, Maryam Farzad

**Affiliations:** ^1^ School of Physical Therapy University of Western Ontario London Ontario Canada; ^2^ School of Rehabilitation Sciences McMaster University Hamilton Ontario Canada; ^3^ Department of Surgery University of Western Ontario London Ontario Canada

**Keywords:** clinical practice guidelines, complex regional pain syndrome, conservative treatment, CRPS, guideline, rehabilitation

## Abstract

**Purpose:**

Several clinical practice guidelines (CPGs) have been developed for complex regional pain syndrome (CRPS). The aim of our study was to appraise CPGs for non‐pharmacological conservative management of CRPS.

**Materials and Methods:**

We systematically searched five electronic databases, from inception to January 2025, to include CPGs that focused on non‐pharmacological conservative management of CRPS. We used AGREE‐II to evaluate the quality of the CPGs. Recommendations, aims, and treatment algorithms of the CPGs were presented in a narrative format, thematic analysis, and matrixes to summarise, categorise, and compare the findings of the guidelines.

**Results:**

Nine CPGs met the inclusion criteria, including three updated versions of previously published guidelines. After accounting for updates, six unique guidelines were appraised. Two were rated as high‐quality, two as moderate‐quality, and two as low‐quality. All CPGs scored > 60% in the AGREE‐II domains of scope/purpose and clarity of presentation, while rigour of development was the lowest‐scoring domain, with only two guidelines achieving ≥ 60%. No guideline addressed updating procedures. The most common interventions recommended by CPGs were pain management (100%) followed by functional restoration (83%), stress‐loading (67%), psychotherapy (67%), edema management (67%), gentle active movements (67%), vocational rehabilitation (67%), normal functional activities (67%), general PT interventions (67%), and isometric‐isotonic strengthening (67%).

**Conclusion:**

The methodological quality of many CPGs for non‐pharmacological management of CRPS is low, particularly in the domain of rigour of development. Recommendations across guidelines are variable, often lacking detail, consistency, and integration of supporting evidence. Pain management, functional restoration, and inter/multidisciplinary care were the most commonly recommended considerations, while guidance on treatment frequency, dosage, and implementation strategies was limited.

**Abbreviations**
AGREE: appraisal of guidelines for research and evaluation
CPGs: clinical practice guidelines
CRPS: complex regional pain syndrome
NSAIDs: non‐steroidal anti‐inflammatory drugs

## 1 Introduction

Complex regional pain syndrome (CRPS), which is a ‘chronic primary pain’ condition, is a rare, debilitating chronic condition that typically develops after a trauma or nerve injury. As the name implies, CRPS is a collection of difficult‐to‐manage signs and symptoms that are always out of proportion to the primary impairment, or trauma [1]. CRPS has a long history of nomenclature changes and the diagnostic criteria for this condition have been changed multiple times over the years. The underlying pathophysiology of CRPS development is still largely unknown [2].

CRPS is not a common condition and various studies have reported a very low prevalence rate [2]. Based on the Nationwide Inpatient Sample database, the largest population‐based study of CRPS in the United States in 2016 revealed that 0.07% of patients (22,533 out of 33,406,123 patients) were discharged with a diagnosis of CRPS [3]. Based on the Truven MarketScan Commercial and Medicare Supplemental databases, another study reported a higher prevalence of 1.2% (78,912 patients out of 6,575,999) [4].

CRPS management has always been challenging for clinicians and the evidence supporting the efficacy of the available treatments is not compelling [5]. Rehabilitation interventions have been the frontline treatment for the management of CRPS over the past two decades [1, 5, 6, 7]. According to the findings from the most recent systematic review and meta‐analysis, there is an overall lack of high‐quality clinical trials. This leads the authors to the conclusion that the evidence on the effectiveness of rehabilitation (non‐pharmacological conservative management) for CRPS is inconclusive [6]. In the absence of clear empirical evidence, expert consensus tends to be favoured in the development of clinical practice guidelines (CPGs) that play an important role in guiding clinicians with their decision‐making.

Over the past two decades, several CPGs have been developed for pharmacological, surgical and rehabilitation management of CRPS [1, 8, 9], though there has yet to be a rigorous systematic synthesis of these guidelines to identify those interventions with the strongest support. In this study, we focused on the recommendations of CPGs for non‐pharmacological conservative (rehabilitative) management of CRPS.

Chronic pain conditions, notably CRPS, are typically associated with physical and psychosocial suffering, both of which aggravate the course of disease. Persistent pain experienced by people with CRPS can lead to avoidance behaviour, fear of movement, decreased mobility, pain catastrophizing, altered functional status and psychological abnormalities. Some authors have opined that this chain of events results in a ‘vicious pain cycle’ that amplifies and prolongs suffering and disability [10, 11].

According to the traditional protocols, the focus of chronic pain management was on the sensory processing of pain instead of considering chronic pain as a multidimensional condition. Therefore, using opioids, NSAIDs, nerve blocks and spinal cord stimulation was more common for the treatment of chronic pain [12]. Over the past two decades, with the upsurge in implementing non‐pharmacological conservative management and rehabilitation interventions, chronic pain conditions have been optimally managed by multidisciplinary interventions to break the vicious pain cycle and address emotional burdens as well as pain, impairment and physical limitations [13, 14].

The aim of our study was to identify, summarise and appraise the CPGs for non‐pharmacological conservative management of CRPS, intended to synthesise CPGs recommendations.

## 2 Methods

The Preferred Reporting Items in Systematic Review and Meta‐Analysis (PRISMA) statement was used to report this systematic review of CPGs [15]. This study was registered on PROSPERO (CRD42023388177). As this was a systematic review, ethical approval was not required.

### 2.1 Search Strategies and Eligibility Criteria

We searched EMBASE, MEDLINE, Google Scholar, PEDro and Cochrane electronic databases, since inception to January 2025. A hand search was also conducted to identify any potential CPG that was not found in the scientific databases. Acknowledging historical shifts in terminology, the search keywords and Boolean operators were: ‘Complex regional pain syndrome OR CRPS OR RSD OR reflex sympathetic dystrophy OR Sudeck's atrophy OR causalgia’ AND ‘conservative OR rehab* OR non‐pharmacolog* OR physiotherap* OR physical therap* OR hand therap* OR occupational therap* OR OT OR PT OR HT’ AND ‘Guideline* OR recommendation OR clinical practice guideline* OR CPG OR review OR systematic review’.

We included CPGs if they mainly focused on non‐pharmacological conservative management of CRPS, or if they provided recommendations on conservative management of CRPS as a part of the guidelines. We included CPGs that were published in English, either in a peer‐reviewed journal or by a panel of experts from national or multinational agencies. However, we did not set any restrictions on the date of publication or the country of origin. Two independent reviewers (ES and MF) ran the literature search and conducted title/abstract screening. In case of any uncertainty about the inclusion of guidelines by title or abstract, the full text of the guidelines was reviewed to check for eligibility. We excluded procedural interventions such as non‐surgical nerve blocks, neuromodulations, injections or combined pharmacology and non‐pharmacological treatments.

### 2.2 Data Extraction and Synthesis

The following data were extracted from the included guidelines: guideline publishers, authors' names, year of publication, country of origin, last update of the guideline, the goal of the guideline, core concepts/themes and the details of the therapeutic pathway or algorithm. The first reviewer (ES) extracted the data from all guidelines and it was double‐checked by the second reviewer (MF).

Data synthesis included extracting and classifying recommendations, providing a narrative summary and identifying consistent recommendations across CPGs. In order to compare, categorise and summarise the interventions recommended by the guidelines, a matrix of guideline recommendations was created and guidelines were marked if they recommended that intervention. Even though the phrasing of each guideline varied, we merged recommendations that were conceptually comparable across guidelines. We used narrative synthesis to describe the aim, scope, main focus and core concepts of the guidelines.

Furthermore, thematic analysis was used in order to identify the aim, scope and theme of the recommendations across CPGs [16, 17]. For the purpose of thematic analysis, the first author assigned a code to each recommendation when extracting data. The general characteristics of the codes to be given were agreed upon before data extraction began, including the main aim of the recommendation (e.g., symptom reduction) or the category of intervention (e.g., routine care or psychotherapy). Then, the second reviewer reread the thematic codes and refined them if required. In the final phase, a group discussion was held and themes were finalised through consensus among all authors.

### 2.3 Quality Appraisal

Two independent reviewers (ES and MF) used the Appraisal of Guidelines for Research and Evaluation (AGREE)‐II [18] to systematically evaluate the quality of the CPGs. Before starting the appraisal, the reviewers conducted calibration exercises on two sample CPGs to ensure consistent interpretation of scoring criteria. Discrepancies between reviewers' scores were resolved through discussion and consensus. If consensus could not be reached, a third reviewer was consulted. The agreement between the two reviewers was quantified using the intraclass correlation coefficient (ICC).

The AGREE‐II comprises 23 items in six domains, including Scope and Purpose (3 items), Stakeholder Involvement (3 items), Rigour of Development (8 items), Clarity of Presentation (3 items), Applicability (4 items) and Editorial Independence (2 items). Each item of the AGREE‐II is scored on a 7‐point Likert‐type scale (1: strongly disagree to 7: strongly agree). Based on the AGREE‐II user's manual, the overall aggregated score is calculated using the following formula: (obtained score – minimum possible score)/(maximum possible score – minimum possible score)*100. The overall quality of the guidelines was judged based on a standardised rubric. A high‐quality guideline is one that has sufficiently covered at least three of the six domains, including ‘rigour of development’ (scored 60% or more on each domain). A moderate‐quality guideline is one with two or more domains, or three domains excluding ‘rigour of development’, rated sufficiently (scored 60% or more on each domain). Guidelines with one or no domain rated sufficiently (scored 60% or more on each domain) were considered low‐quality [18, 19].

Although the AGREE‐II instrument was published after some of the included guidelines, all CPGs—regardless of publication date—were appraised using AGREE‐II to allow a standardised comparison across documents. We acknowledge that applying AGREE‐II to older guidelines may influence the interpretation of certain domain scores, particularly the rigour of development.

In order to visually illustrate and compare the domain‐level quality of guidelines, a polar bar chart was created using the Vizzlo website (https://vizzlo.com).

## 3 Results

### 3.1 Literature Search

**Figure 1:**
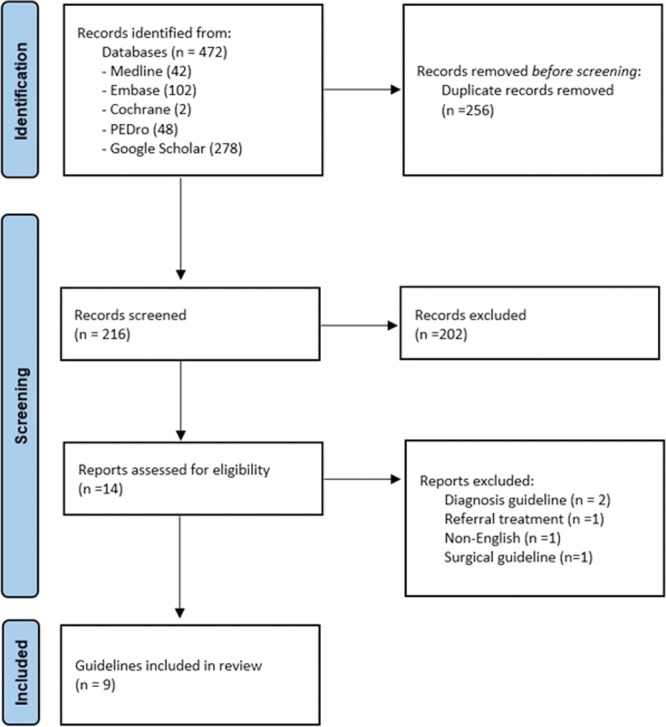
Prisma flow diagram of the included guidelines.

Our search strategy yielded a total of 472 records. After deduplication and rigorous title/abstract checking by the two reviewers, 14 records were deemed eligible for full‐text review, of which nine CPGs met the inclusion criteria [9, 20, 21, 22, 23, 24, 25, 26]; however, three were updated versions of previously published guidelines, resulting in six unique guidelines being analysed in this review. The five excluded records [27, 28, 29, 30, 31] were guidelines on the diagnosis and surgical procedures for the management of CRPS (Figure 1).

### 3.2 Characteristics of Included Guidelines

Nine CPGs that included non‐pharmacological conservative management of CRPS were published between 1998 and 2022 [9, 20, 21, 22, 23, 24, 25, 26]. There was only one CPG that specifically focused on non‐pharmacological conservative management for CRPS [26]. The other guidelines provided recommendations on diagnosis, referral, rehabilitation interventions, psychotherapy and pharmacological/surgical management of CRPS. (Table 1).

**Table 1:** An overview of the clinical practice guidelines.

Author/Publisher, year	Country of origin	Quality (AGREE‐II)	Aim/Scope
Stanton‐Hicks, 1998 [9] ‎	USA	Low	Present an orderly approach for the treatment of chronic CRPS‐I and ‐II.
Stanton‐Hicks, 2001 [20]	USA	Low	Incorporating interdisciplinary approaches and refining the linear approach of timing and sequencing of the treatments proposed in the previous guideline.
Perez S, 2010 [21]‎	Netherlands	High	Developing evidence‐based multidisciplinary guidelines for treatment of CRPS‐I from studies published between 1980 to June 2005.
Goebel, 2011, 2012, 2018 [32]	UK	High	Guidelines for diagnosis, referral and management of CRPS were developed by an expert panel and are based on the experts' opinions.
Norman ‎‎2013, 2022 [23, 24]	USA	Moderate	Developed by a collaboration of expert practitioners and adapted from three expert consensus meetings: Malibu (1987), Minneapolis (2001) and Budapest (2004).
Goebel, 2019 [26]	A collaboration of 37 countries in Europe	Moderate	Developed by a collaboration between the experts of the European Pain Federation and CRPS patients. Presenting 17 standards in eight areas for use by healthcare providers, commissioners and policymakers for the purpose of identification and appropriate resource allocation.

One of the records was a concise guidance adopted from the detailed guidelines published by the UK Royal College of Physicians [25]. Two of the CPGs had published an update [22, 24] on their previous versions [22, 23]; however, the recommendations were the same in both the older and updated versions. This reduces the number of analysed CPGs to six. Three of the CPGs were published in the United States [9, 20, 24], one in the Netherlands [21], one in the United Kingdom [22] and one was a collaboration between 37 European countries [26]. A single guideline used exclusively peer‐reviewed empirical evidence [21], while the others were based mostly on expert opinions.

A total of 47 recommendations, interventions and techniques were identified by the CPGs. The most common interventions recommended by CPGs were pain management (100% of the CPGs) followed by functional restoration (83%), stress‐loading (67%), psychotherapy (67%), edema management (67%), gentle active movements (67%), vocational rehabilitation (67%), normal functional activities (67%), general PT interventions/modalities (67%), isometric‐isotonic strengthening (67%).

Through thematic analysis, each of these 47 recommendations was given a specific code by the first author. A total of 12 codes (subthemes) were assigned to the recommendations in the first round. The second reviewer reread the codes and merged a few codes, resulting in a total of 7 codes (subthemes) in the second round. Through consensus between the two reviewers and a group discussion among authors in the third round, a total of three general themes were assigned to the recommendations of CPGs, including symptom reduction, functional restoration and inter/multidisciplinary care.

### 3.3 Quality Appraisal

**Figure 2:**
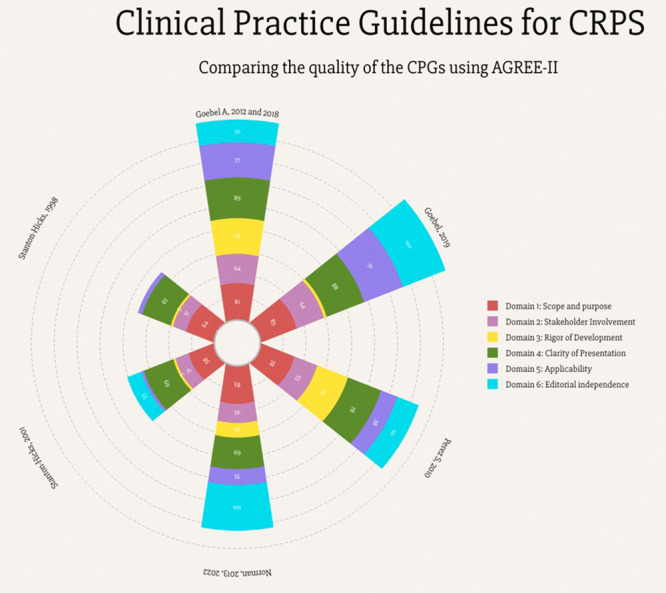
Agree‐II scores for CPGs.

The agreement between the two raters in quality appraisal was high (ICC > 0.90). Appraising CPGs using AGREE‐II indicated that two CPGs were rated as high‐quality [21, 22], two as moderate‐quality [24, 26] and two as low‐quality [9, 20]. All CPGs adequately addressed two AGREE‐II domains (scope/purpose and presentation clarity) (scored > 60%). Rigour of development was the lowest scoring domain, with only two guidelines [21, 22] scored 72% and 78%, while the other guidelines scored less than 40%. The only item that none of the guidelines considered was updating the plan and procedure, while three guidelines (out of nine) were an update of an older version. Only two CPGs scored high on search method and literature search [21, 22]. Details of the results of the quality appraisal are presented in Table 2 and Figure 2.

**Table 2:** Appraisal of guidelines, research and evaluation of the guidelines AGREE‐II.

Agree‐II domains	Stanton‐Hicks, 1998 [9]	Stanton‐Hicks, 2001 [20]	Perez S, 2010 [21]‎	Goebel A, 2011, 2012 and 2018 [32]	Norman, ‎‎2013, 2022 [23, 24]	Goebel, 2019 [26]
Scope/Purpose	64	58	78	81	83	83
Stakeholder involvement	31	31	53	64	42	64
Rigour of development	5	5	72	78	32	6
Clarity of presentation	67	69	78	89	69	88
Applicability	10	6	38	77	35	91
Editorial independence	0	33	50	50	100	100
Overall quality	Low	Low	High	High	Moderate	Moderate

### 3.4 Summary of the CPGs Findings

CPGs elaborated different approaches to propose a step‐by‐step clinical pathway for the conservative management of CRPS. However, the majority of them agreed on early referral to rehabilitation as the anchor for optimal management. Two components were identified as the core of all the CPGs, including symptom reduction and functional restoration. Developing a strong therapeutic rapport with patients, keeping them motivated and engaged in therapy and patient education were pointed as the keys to successful treatment.

The majority of the recommendations focused on pain management, functional restoration, multi/interdisciplinary care and a gradual increase in the intensity of the interventions. In case of failing to progress through the treatment algorithm within a specific period of time (1–3 months), more intensive rehabilitation interventions (in terms of frequency and dosage), pharmacotherapy, psychotherapy, behavioural therapy, or referring to specialised care for pain management were usually recommended. More details on the summary of each CPG are provided in Table 3.

**Table 3:** Summary of findings.

Author, year	Guideline development	Summary of findings
Stanton‐Hicks, et al. 1998 [9]	A consensus treatment guideline to present an orderly approach for the treatment of chronic CRPS I and II.	Central theme → Functional restoration. Two essential components → Self‐management and functional rehabilitation.
		Core concepts → Motivation, mobilisation and desensitisation.
		This guideline includes four steps: 1. Developing therapeutic alliance and rapport. 2. Incorporating motivation, mobilisation and desensitisation. 3. Isometric strengthening and stress‐loading. 4. Completing functional recovery.
		Last stages of the algorithm→ Self‐management and minimising dependence on invasive and technical modalities.
Stanton‐Hicks, 2002 [20]	Incorporating interdisciplinary approaches and refining the linear approach of timing and sequencing of the treatments proposed in the previous guideline.	The goal of treatment → Minimisation of pain and optimisation of function through rehabilitation, pain management and psychological therapy. Not responding to treatment within 12–16 weeks →More interventional therapies. Successive steps in the pathway →Achieving gentle active ROM, stress loading, scrubbing techniques, isotonic strengthening, general aerobic conditioning and postural normalisation. Key to successful treatment → Keep patients motivated and engaged. Failure to progress in each stage →Stronger drugs for pain relief, more intensive psychotherapy, or the use of more aggressive pain management techniques, such as regional anaesthesia or SCS.
		This guideline includes three stages: 1. Developing a strong therapeutic alliance and rapport for a successful occupational and physical therapy intervention. 2. Increase patients' flexibility with the use of gentle active ROM. 3. Normalising use of the affected limb, assessment of ergonomics, posture and required modifications at home and the workplace and complementary recreation therapy and vocational rehabilitation.
Perez S, et al. 2010 [21]	Developing evidence‐based multidisciplinary guidelines for treatment of CRPS‐I from studies published between 1980 to June 2005.	Recommendations based on the level of evidence: 1. Level‐2 evidence → Physiotherapy (in general) for upper limb CRPS‐I is likely to have a positive impact on this condition and patients' coping skills. 2. Level‐3 evidence → Physiotherapy interventions might be effective for the treatment of chronic CRPS‐I. 3. Level‐4 evidence → Physiotherapy should be included in the routine treatment of CRPS‐I. 4. Level 4 evidence → Insufficient evidence for the effectiveness of TENS in the treatment of CRPS‐I. 5. Level‐3 evidence → OT could be effective in functional and activity limitation improvement. 6. Level‐4 evidence → No evidence on the effectiveness of multidisciplinary treatment of CRPS‐I.
Goebel A, et al. 2012 [32]	Extracted and adapted from the UK guidelines for diagnosis, referral and management of CRPS developed by an expert panel and based on experts' opinions.	Four pillars of treatment for CRPS: 1. Patient information and education 2. Pain relief 3. Physical and vocational rehabilitation 4. Psychological interventions
		General Recommendations: 1. Early diagnosis to prevent secondary physical limitations and psychological consequences of undiagnosed chronic pain. 2. To avoid symptoms' progression, → Early referral to PT and start of gentle active movements. 3. Referring more severe cases to a pain management specialist and rehabilitation programmes. 4. Paying attention to an integrated interdisciplinary treatment approach. 5. Providing patient education about CRPS. 6. Complex CRPS → Having access to specialist interdisciplinary rehabilitation programmes. 7. Close teamwork between specialist rehabilitation teams and pain management services. 8. Access to rehabilitation medicine care in the context of a cognitive behavioural approach involving both the patient and their family. 9. Features of the rehabilitation programme → Being goal‐oriented and actively engaging patients and their family in goal setting to make patients responsible for the rate of progress. 10. Early access to vocational assessment.
		Rehabilitation treatment algorithm**:** *CRPS patients with mild/moderate‎ symptoms* 1. Patient education + routine treatment. 2. Successful treatment and an ongoing improvement should be evident within 4 weeks. → Failing to respond to routine treatment or for patients with moderate/severe symptoms and/or dystonia. 1. Referring to multidisciplinary pain clinics and more specific treatments^+^.
Harden R. Norman, et al., 2013, 2019 [23, 24]	Developed by a collaboration of expert practitioners (evidence‐based + expert opinion). Adapted from three expert consensus meetings: Malibu (1987), Minneapolis (2001) and Budapest (2004).	Overall rehabilitation treatment algorithm in 4 steps: 1. Mirror visual feedback (MVF), Graded motor imagery (GMI), Reactivation, Contrast baths, ‎Desensitisation and Exposure ‎therapy. 2. Oedema control, Flexibility (active), Isometric strengthening, Correction ‎of postural abnormalities and Diagnosis and treatment of secondary ‎myofascial pain. 3. Stress loading, Isotonic strengthening, ROM (gentle, passive), ‎General aerobic conditioning, Postural normalisation and balanced ‎use. 4. Ergonomics, Movement therapies, Normalisation of Use and ‎Vocational/Functional Rehabilitation ** Failing to start or progressing through the treatment algorithm→More or stronger medications, more intensive psychotherapies and/or more interventional therapies.
Andreas Goebel, 2019 [26]	Developed by a collaboration between the experts of the European Pain Federation and CRPS patients, including 17 standards in 8 areas.	Covered areas: Diagnosis, multidisciplinary care, assessment, care pathways, information and education, pain management, physical rehabilitation and distress management*Diagnosis*→ 1. Using the Budapest criteria for diagnosis. 2. No use of diagnostic tests, except for excluding other diagnoses. *Multidisciplinary care*→ 3. The severity of CRPS is the indicator of the need for multidisciplinary care for more severe and complex cases. *Assessment*→ 4. A thorough evaluation identifying any triggers for the CRPS, the severity of pain and how it affects the ability to function, engagement in ADL, participation in extracurricular activities, quality of life, sleep and mood regulation. *Care pathway*→ 5. Referring to specialised care in case of no improvement in pain and function within two months after starting treatment, despite good adherence to treatment. 6. Cues for referral to super‐specialised care→ CRPS spread, fixed dystonia, myoclonus, skin ulcerations or infections or malignant oedema in the affected limb and extreme psychological distress. 7. Advanced CRPS treatments, such as multidisciplinary psychologically informed rehabilitation pain management programmes (PMP), must be made available in specialised care facilities. *Information and education*→ 8. Providing adequate information to patients, their dependents, or carers, regarding CRPS causation, natural course, signs and symptoms, outcomes and treatment options by therapeutic disciplines. *Pain management*→ 9. Accessing pharmacological treatments appropriate to CRPS or other similar neuropathic pain conditions. 10. Pain management must be accompanied by a tailored rehabilitation plan. 11. Stopping guidelines and a medication reduction plan in case of demonstrating adverse events or non‐efficiency. 12. Reassessment of patients due to the alteration of the clinical picture of CRPS. *Physical rehabilitation*→ 13. Early and frequent assessment in terms of affected limb function, general body function and home/work/school activity participation, as appropriate. Vocational rehabilitation should be offered, as needed. 14. Early referral to OTs and PTs. 15. Available training on basic approaches of CRPS pain management for OTs and PTs. *Distress management*→ 16. Depression, anxiety, post‐traumatic stress, pain‐related fear and avoidance should be assessed as distress factors. 17. Evidence‐based psychological treatment should be available to CRPS patients, as needed.

## 4 Discussion

To the best of our knowledge, this study is the first systematic review of CPGs for non‐pharmacological conservative management of CRPS. We found six original guidelines published between 1998 and 2022. There were only two low‐quality CPGs [9, 20] that were published in 1998 and 2002. The other CPGs were of high‐ and moderate‐quality. The majority of CPGs were based on expert opinions. In the absence of compelling empirical evidence, it is impossible to report the anticipated effectiveness of most interventions reported in the CPGs and most guidelines were developed by national professional associations or experts, meaning some recommendations might not be applicable to other nations or cultural contexts [9, 33].

In most CRPS CPGs, rehabilitation interventions were introduced as the mainstay and core of CRPS management [9, 20, 23, 24]. Since one of the defining characteristics of CRPS is the variability of presentations between patients as well as the wide range of signs and symptoms experienced by a given patient [8], CPGs were mostly in favour of implementing multimodal and multidisciplinary care for the conservative management of CRPS [34, 35]. However, empirical evidence is limited to single treatments rather than multimodal therapies as a whole.

Since CRPS affects multiple aspects of a person's health, patients with CRPS typically have functional limitations that prevent them from working or participating in social activities. Due to the multifactorial and enigmatic nature of CRPS onset and development, a successful treatment approach should be one that covers multiple dimensions and mechanisms of this complex condition and the patient's health [7]. Neither a single‐treatment nor an all‐treatment approach should be suggested for the optimal management of CRPS. According to the recommendations of the previous empirical studies [8], the ideal treatment method is the one that takes a holistic approach and does not prioritise impairment or body function/structures over the contributions of environmental and personal factors [36]. Improving participation in activities of daily living and therapy should also be regarded as one of the therapeutic targets, as it has been in some CPGs through multimodal and interdisciplinary care [26]. Vocational rehabilitation, recreation therapy, ergonomics, goal setting and sleep hygiene‎ are some of the examples of consideration for participation at the latter stages of the treatment algorithm.

Increasing patient motivation to increase patient engagement and adherence to treatment [9, 20, 23, 24] was also one of the factors that was recommended. However, no specific strategy has been proposed to increase patient motivation. Although no evidence was found for patient motivation as a treatment approach, three guidelines emphasised patient motivation as one of the pillars of successful treatment [9, 20, 23, 24]. Future research and investigations should delve into potential strategies or interventions aimed at cultivating and sustaining patient motivation within the context of the discussed treatments. Exploring the nuanced approaches required to effectively bolster patient motivation is essential for improving patient outcomes.

The pathophysiological mechanisms underlying the development, progression and maintenance of CRPS remain poorly understood and it presents a complex interplay of various pathophysiological processes, leading to heterogeneous clinical presentations observed among patients. Recognising this complexity, researchers and clinicians are increasingly focusing on developing treatment strategies that target these specific underlying mechanisms rather than solely managing symptoms. Increased understanding of the pathogenesis of CRPS has provided the opportunity to develop a shift towards mechanism‐based treatments [37, 38]. Despite this shift, current evidence and CPGs often lack specific guidance on mechanism‐based interventions, highlighting a gap in knowledge and practice. As our understanding of the pathogenesis of CRPS deepens, there is a growing opportunity to develop and evaluate mechanism‐based treatments tailored to the individual profiles of CRPS patients. This shift towards personalised, mechanism‐driven interventions holds the potential to significantly improve outcomes for individuals grappling with the challenges of CRPS [38]. Future research endeavours should aim to illuminate the effectiveness and feasibility of mechanism‐based interventions within the CRPS population, paving the way for enhanced clinical care.

In order to appraise the quality of CPGs, we used the AGREE‐II instrument, which was published in 2009 [18]. We should note that CPGs that were published prior to 2009 [9, 20] have been judged against a tool that was developed later and might have been underrated in quality appraisal, while the CPGs that were published after 2009 received higher ratings for quality assessment. However, the findings of low‐quality guidelines do not differ from those of moderate‐ and high‐quality guidelines. ‘Rigour of Development’ was one of the AGREE‐II domains that was given priority over other domains in assigning an overall rating for a CPG. This domain investigates literature search, evidence‐based recommendations and the link between recommendations and evidence. Since there were only two guidelines [21, 22] that integrated evidence‐based findings with expert opinion, the other guidelines were rated poorly (less than 40%) in this domain.

A number of disparities were evident when comparing the findings of CPGs with the most recent systematic reviews and meta‐analyses on conservative management of CRPS [5, 6]. In the most recent systematic review by Shafiee et al. [6], the highest quality evidence (moderate‐quality) was in favour of employing movement representation techniques, such as mirror therapy and graded motor imagery programmes. In contrast, movement representation techniques were only recommended in two of the CPGs [22, 23, 24, 25] reviewed here. Notwithstanding the evidence, electrical stimulations were recommended in 4/6 of the CPGs [20, 21, 22, 23, 24, 25], while systematic reviews and meta‐analyses have previously found this is not an effective intervention [5, 6]. Pain management and functional restoration, on the other hand, were the most commonly proposed intervention plans in CPGs; however, specific therapeutic interventions, dosage of treatment and a detailed strategy to address these outcomes were not provided.

These discrepancies create substantial uncertainty for clinicians attempting to practice evidence‐based medicine. The American Academy of Pain Medicine guidelines acknowledge this ‘evidence vacuum’ explicitly, noting that practitioners must treat patients despite limited high‐quality research, often extrapolating from related conditions. As a result, clinicians are frequently compelled to rely on clinical judgement and empirical experience rather than robust evidence. The mismatch may also lead to suboptimal resource allocation. Invasive neuromodulation interventions require specialised centres, thorough psychosocial evaluation and substantial costs for device implantation and maintenance, yet rest on very low‐certainty evidence. Meanwhile, sensorimotor approaches with stronger evidence may be underutilised, partly because they demand significant time and patient commitment, which can limit practical implementation.

The heterogeneity in guideline recommendations reflects fundamental gaps in understanding CRPS pathophysiology. Without clear therapeutic targets, treatment selection lacks a strong mechanistic rationale, contributing to the poor treatment responses commonly observed in CRPS. These discrepancies likely arise from delays in updating guidelines, reliance on expert consensus and historical practice and limited clarity regarding disease mechanisms. Consequently, clinicians may continue using low‐certainty interventions while underutilising evidence‐supported therapies, leading to inconsistent care, inefficient resource allocation and potentially suboptimal patient outcomes. This incongruity poses critical questions for clinical practice and future research directions. For clinical practice, it suggests the potential need to reevaluate the recommendations provided in the CPGs regarding the efficacy of certain interventions. It highlights the importance of basing clinical decisions on the most up‐to‐date and highest‐quality evidence available, such as that provided by systematic reviews and meta‐analyses. In terms of research implications, these findings underscore the necessity of ongoing scrutiny of the evidence‐based supporting treatment guidelines. Future research efforts should focus on reconciling these discrepancies, possibly through well‐designed comparative effectiveness studies. Additionally, there is a need for more comprehensive reporting within CPGs, including specific therapeutic interventions, treatment dosages, and detailed strategies to address pain management and functional restoration outcomes. Addressing these disparities will be vital for ensuring that clinical practice aligns with the best available evidence, ultimately leading to improved outcomes for patients with CRPS.

In the past decade, the establishment of an international consortium under the auspices of the International Association for the Study of Pain (IASP) CRPS Special Interest Group (SIG) to develop the core outcome measures for CRPS clinical trials (COMPACT) marked a significant advancement [39]. This initiative aims to create a core set of outcome measures for CRPS clinical trials. The COMPACT initiative holds the promise of facilitating more comprehensive and evidence‐based studies in the future. It seeks to address the incongruities apparent in research and practice related to CRPS by forming a large international cohort.

Lack of high‐quality evidence is one of the main paucities of the literature on non‐pharmacological conservative management for CRPS [5, 6]. A number of potential causes have been identified for this paucity. One of them could be that CRPS is not a common condition and most of the RCTs are of low quality due to small sample sizes in clinical research studies. Furthermore, CRPS is characterised by fluctuating signs and symptoms and it most likely requires a more personalised approach to care. Therefore, due to the heterogeneity of CRPS, recruiting a homogenous sample of patients could be challenging. Moreover, CRPS diagnosis has been challenging, and diagnostic criteria have been changed several times over the past two decades, which could lead to the misdiagnosis or overdiagnosis of CRPS patients [40]. While the diagnostic criteria have changed, the most recent two versions are the ‘Budapest criteria’ (2010) and the subsequent ‘Valencia’ revision (2019) [41, 42, 43]. The Valencia revision aligns some terminology for CRPS with the International Classification of Diseases (ICD‐11) and made adaptations to the diagnostic taxonomy text.

## 5 Conclusion

Pain management, functional restoration and multi/interdisciplinary care are the most frequently recommended strategies in CPGs for non‐pharmacological conservative management of CRPS. However, the guidelines exhibit low methodological quality, especially regarding rigour of development and often provide inconsistent or insufficiently detailed recommendations. Applicability and implementation guidance are limited, reducing their practical utility. Moving forward, efforts to improve guideline quality should focus on transparent evidence appraisal, standardised reporting of interventions and dosage, and enhanced guidance on implementation. Additionally, adaptive trial designs, collaborative research efforts, patient registries, and standardised outcome measures may support future evidence‐based refinement of multimodal CRPS management.

## Funding

The authors have nothing to report.

## Conflicts of Interest

The authors declare no conflicts of interest.

## Data Availability

The authors have nothing to report.
